# A Biomarker to Differentiate between Primary and Cocaine-Induced Major Depression in Cocaine Use Disorder: The Role of Platelet IRAS/Nischarin (I_1_-Imidazoline Receptor)

**DOI:** 10.3389/fpsyt.2017.00258

**Published:** 2017-12-01

**Authors:** Benjamin Keller, Joan-Ignasi Mestre-Pinto, María Álvaro-Bartolomé, Diana Martinez-Sanvisens, Magí Farre, M. Julia García-Fuster, Jesús A. García-Sevilla, Marta Torrens, The NEURODEP Group, F. Fonseca

**Affiliations:** ^1^Laboratori de Neurofarmacologia, IUNICS, Universitat de les Illes Balears (UIB), Fundació Institut d’Investigació Sanitària Illes Balears (IdISBa), Palma, Majorca, Spain; ^2^Redes Temáticas de Investigación Cooperativa en Salud – Red de Trastornos Adictivos (RETICS-RTA), Instituto de Salud Carlos III (ISCIII), Madrid, Spain; ^3^Hospital del Mar Medical Research Institute (IMIM), Institut de Neuropsiquiatria i addiccions (INAD), Barcelona, Spain; ^4^Hospital Universitari Germans Trias i Pujol (IGTP), Badalona, Spain; ^5^Universitat Autònoma de Barcelona (UAB), Barcelona, Spain

**Keywords:** platelet biomarker, IRAS/nischarin, cocaine use disorder, major depressive disorder, cocaine-induced depression, acute tryptophan depletion, antidepressant drugs

## Abstract

The association of cocaine use disorder (CUD) and comorbid major depressive disorder (MDD; CUD/MDD) is characterized by high prevalence and poor treatment outcomes. CUD/MDD may be primary (primary MDD) or cocaine-induced (CUD-induced MDD). Specific biomarkers are needed to improve diagnoses and therapeutic approaches in this dual pathology. Platelet biomarkers [5-HT_2A_ receptor and imidazoline receptor antisera selected (IRAS)/nischarin] were assessed by Western blot in subjects with CUD and primary MDD (*n* = 16) or CUD-induced MDD (*n* = 9; antidepressant free, AD−; antidepressant treated, AD+) and controls (*n* = 10) at basal level and/or after acute tryptophan depletion (ATD). Basal platelet 5-HT_2A_ receptor (monomer) was reduced in comorbid CUD/MDD subjects (all patients: 43%) compared to healthy controls, and this down-regulation was independent of AD medication (decreases in AD−: 47%, and in AD+: 40%). No basal differences were found for IRAS/nischarin contents in AD+ and AD− comorbid CUD/MDD subjects. The comparison of IRAS/nischarin in the different subject groups during/after ATD showed opposite modulations (i.e., increases and decreases) in response to low plasma tryptophan levels with significant differences discriminating between the subgroups of CUD with primary MDD and CUD-induced MDD. These specific alterations suggested that platelet IRAS/nischarin might be useful as a biomarker to discriminate between primary and CUD-induced MDD in this dual pathology.

## Introduction

The association of substance use disorders and comorbid major depressive disorder (MDD) is characterized by high prevalence rates (12–80%) (1) and challenging clinical management of patients, implying a great burden for health care systems (2). In particular, a robust prevalence (27–34%) has been reported in previous studies (3, 4) for the co-morbidity of MDD and cocaine use disorder (CUD), in which MDD can be independent (primary MDD) or cocaine-induced (CUD-induced MDD). In this context, the distinction between independent and CUD-induced MDD might be crucial to improve treatment strategy and outcomes (5). To date, the diagnosis is based on clinical criteria [using DSM-5 (6) or ICD-10 (7)] and there is a need for specific biomarkers to facilitate the differentiation between primary MDD and CUD-induced MDD to improve diagnosis and clinical management. In this sense, there is a growing research about putative biomarkers, mainly involving cytokines, chemokines, several fatty acid derivatives such as endocannabinoids and neurotrophic factors such as brain-derived neurotrophic factor, insulin-like growth factor-1-binding protein 3 (8–10).

Other potential biomarkers for MDD and/or CUD include the platelet serotonin 5-HT_2A_ receptor (11, 12) and the I_1_-imidazoline receptor (I_1_-IR) candidate IR antisera selected (IRAS)/nischarin (13–16). Thus, serotonin abnormalities have been implicated in the pathogenesis of MDD and platelet 5-HT_2A_ receptors, as a peripheral parameter to indirectly measure brain serotonergic function, has been routinely assessed as a biomarker in a number of studies [e.g., see Ref. (11), which combines platelet 5-HT_2A_ receptor binding and plasma tryptophan/amino acid ratio in MDD]. Notably, the I_1_-IR was recently shown to participate in the prevention of cue-induced cocaine relapse in rats (17) and has been proposed as a state marker of MDD (18, 19). In a recent post-mortem human brain study, the basal content of I_1_-IR was marginally increased and then downregulated by antidepressant drugs (AD) in the prefrontal cortex of subjects with MDD (15), further suggesting a role of this novel receptor in MDD and in the action mechanisms of AD, including the selective 5-HT re-uptake inhibitors (SSRI) which were the predominant drugs in the medicated depressed patients of the present study.

Based on dysregulated (decreased) 5-HT transmission in mood disorders such as MDD, the depletion of the 5-HT precursor tryptophan (Trp) was introduced as a useful tool to study the involved disease mechanisms and potential biomarkers affected by reduced 5-HT synthesis and bioavailability (20–22). Thus, the aim of the present study was to assess two potential biomarkers: 5-HT_2A_ receptor and IRAS/nischarin, in platelets of patients with comorbid CUD and primary- or CUD-induced MDD at basal level and after acute tryptophan depletion (ATD) test (22).

## Materials and Methods

### Subjects and Recruitment

Patients included in the study were recruited at the Addiction treatment facilities of the Institute of Neuropsychiatry and Addiction of Parc de Salut Mar, in Barcelona. A total of 110 patients were informed about the study. Of them, 23 patients refused to participate, 36 patients did not meet inclusion criteria, and 26 patients did not complete the study. Healthy controls were healthy volunteers, were recruited from a database of healthy subjects willing to participate in medical research projects. For this study, some subjects were postgraduate university students. Thus, a total of 35 subjects participated in the study; 16 cases diagnosed of CUD and primary MDD, 9 cases diagnosed of CUD and CUD-induced MDD, and 10 healthy controls (Table 1). All diagnoses were done according DSM-IV-TR criteria (23). Inclusion criteria included both genders, older than 18 years, Caucasian origin, and body mass indices between 19 and 29 kg/m^2^. In the MDD groups (primary or CUD-induced), the most recent episode should be in remission, and Hamilton Depression Rating Scale (HDRS) (24) scores should be less than 6. In the CUD groups, subjects should maintain at least 4 weeks of abstinence, confirmed by negative urine controls. Exclusion criteria included: cognitive or language limitations that precluded evaluations; pregnant or breastfeeding women and any medical problem that could interfere in the study procedures; in the comorbid CUD/MDD groups: any psychiatric disorder in Axis I (DSM-IV-TR) other than MDD, and/or any substance use disorders other than cocaine or nicotine use disorder; in the healthy control group: any psychiatric disorder in Axis I (DSM-IV-TR), family history of depressive disorder and any substance use disorders (except nicotine use disorder).

**Table 1:** Sociodemographic and clinical characteristics of the various groups of subjects.

	CUD/MDD, *n* = 25		
	Primary MDD, *n* = 16	Induced MDD, *n* = 9	Healthy controls, *n* = 10
Sex (M/F)	12/4	7/2	5/5
Age (mean, years)	45 ± 2	38 ± 4	34 ± 2
**Depression**			
Age of onset (years)	36 ± 3	33 ± 4	
Number of episodes	2.3 ± 0.3	6.2 ± 2.6	
Age at last episode	42 ± 2	35 ± 4	
Months since last episode (remission)	23 ± 10	30 ± 12	
Family history of MDD	10 (62.5%)	4 (44.4%)	
Current antidepressant treatment	11 (68.8%)^a^	4 (44.4%)^b^	
**Tryptophan depletion test**			
plasma Trp/LNAA (μmol/l) basal	0.08 ± 0.009	0.08 ± 0.01	0.07 ± 0.02
plasma Trp/LNAA (μmol/l) at 5 h	0.008 ± 0.002	0.007 ± 0.001	0.007 ± 0.001

*Data are mean ± SEM values (*n*, number of subjects). CUD subjects: at least 1 month of cocaine abstinence; MDD subjects were in remission of MDD symptoms (Hamilton Depression Rating Scale, score <6). CUD, cocaine use disorder; MDD, major depressive disorder; M, male; F, female; Trp, tryptophan; LNAA, large neutral amino acids*.

*^a^Antidepressant treatments: selective serotonin re-uptake inhibitors (SSRI, *n* = 6), SSRI + mirtazapine (*n* = 2), ISSRI + trazodone (*n* = 1), venlafaxine (*n* = 1), and venlafaxine + trazodone (*n* = 1)*.

*^b^Antidepressant treatments: mirtazapine (*n* = 2), trazodone + SSRI (*n* = 1), and venlafaxine (*n* = 1)*.

The clinical protocol was approved by the local Research Ethical Committee (CEIC-Parc de Salut Mar, Barcelona, Spain) and the study was conducted in accordance with the Declaration of Helsinki and Spanish laws concerning clinical research. Healthy controls were financially compensated. All subjects gave the written, informed consent prior to participation in the study. The neurochemical protocol was also approved by the Ethics Committee of Clinical Investigation (CEIC-CAIB) and developed following the guidelines of the University of the Balearic Islands (UIB).

### Clinical Assessments

A close-ended questionnaire was used to record patients’ sociodemographic characteristics, family history, medical assessment including serological status (human immunodeficiency virus and hepatitis C virus) history of substance use and previous psychiatric treatment. Depression severity was evaluated with the Spanish version of the HDRS (25).

### Psychiatric Research Interview for Substance Use and Mental Disorders (PRISM)

Substance and non-substance use disorders were diagnosed according to DSM-IV-TR criteria (23), using the Spanish version of the PRISM-IV (26).

The PRISM is a semi-structured interview designed to evaluate current and life-time DSM-IV-TR disorders and has showed good to excellent validity and test–retest reliability for primary (MDD) and substance-induced major depression with kappa ranging from 0.66 to 0.75 (26, 27).

### Acute Tryptophan Depletion Test

Patients and healthy controls participated in the ATD test (22), which was performed in a randomized, double-blind, within-subject, crossover and placebo-controlled design. Prior to the study a validation and standardization of the ATD test was performed in healthy controls. The two experimental sessions were conducted with at least a 1-week washout period between them (ATD and non-ATD sessions). In female subjects, the menstrual cycle was controlled and the first session was conducted during the follicular phase to control possible differences in the results due to the involvement of the serotonergic system in hormonal changes.

For the 24 h prior to each session, subjects consumed a low Trp diet (22) and they were fasting on the day of the laboratory sessions. Before starting the sessions, urine was screened for cocaine and other substances to exclude consumption prior to testing. During one session, in randomized form, the subjects were given an amino acid mixture lacking Trp (ATD session; i.e., l-alanine 4.1 g, l-arginine 3.7 g, l-cysteine 2.0 g, glycine 2.4 g, l-histidine 2.4 g, l-isoleucine 6.0 g, l-leucine 10.1 g, l-lysine 6.7 g, l-methionine 2.3 g, l-proline 9.2 g, l-phenylalanine 4.3 g, l-serine 5.2 g, l-threonine 4.9 g, l-tyrosine 5.2 g, and l-valine 6.7 g) and in the other session a similar amino acid mixture but containing Trp (non-ATD; 3.0 g) (19). Blood samples were obtained prior, during and after the test. With this technique, free and total plasma Trp levels are markedly reduced 3–5 h after the depleting Trp drink. A Trp-enriched diet (high Trp/large neutral amino acids, LNAA, ratio), given at 6 h led to the recovery of plasma Trp to basal levels at 24 h (22).

### Blood/Platelet Samples

During both sessions, venous blood was obtained by inserting a venous catheter in the non-dominant hand from healthy volunteers (*n* = 10) and CUD/MDD patients (*n* = 25, Table 1) before starting the test (basal), at maximum plasma Trp depletion (5 h after induction) and at recovery of plasma Trp levels (24 h after induction) (see Table 1). At each time, aliquots of 5 ml blood from each subject were collected in plastic tubes containing citrate-dextrose solution (ACD, 0.06 mol/l sodium citrate, 0.38 mol/l citric acid, and 0.136 mol/l dextrose) as anticoagulant (8:1 vol/vol). Platelets were isolated by centrifugation (350 × *g* for 15 min at room temperature) on 5 ml of one-step Human Platelets™ Cell Separation Medium (5 mmol/l Tricine/sodium hydroxide buffer, pH 7.0, containing 12% NycodenzR, and 0.56% sodium chloride; Nycomed Pharma AS, Oslo, Norway). Then, the translucent platelet layer was recovered, mixed with 2 vol of 0.9% NaCl, and recentrifuged (600 *g* for 5 min) to pellet the platelets that were stored at −80°C until the biochemical assays.

### Platelet Membrane Preparation

Platelet samples were washed in 500 µl of 0.9% NaCl, isolated by centrifugation (3,000 × *g* for 15 min at 4°C) and prepared (with minor modifications) for Western blot analysis as described (28, 29). Briefly, the pellet was sonicated in 1 ml of 50 mmol/l Tris buffer, pH 7.5, containing 1 mmol/l ethylenediaminetetracetic acid, 2 mmol/l magnesium chloride (MgCl_2_), as well as 10 µl/ml of protease and phosphatase inhibitor cocktails (P8340 and P2850; Sigma-Aldrich). The suspension was centrifuged (16,000 × *g* for 30 min at 4°C), the resulting pellet (platelet membranes) thoroughly resuspended in protease/phosphatase inhibitor-free Tris buffer. Then, 60 µl of 160 mmol/l Tris, pH 6.8, with 8% sodium dodecyl sulfate (SDS) were added and the mixture heated for 5 min, followed by 5 min sample chilling on ice. Bicinchoninic acid assay was used to determine the sample protein concentration, which was adjusted to 3–4 µg/µl with protease/phosphatase inhibitor-free Tris buffer. Finally, the platelet samples were mixed (9:1, vol/vol) with electrophoresis loading buffer (300 mmol/l Tris, pH 6.8, 20% SDS, 70% glycerol, and 0.03% bromophenol blue) and boiled for 3 min.

### Immunoblot Assays and Quantification of Target Protein Contents

The contents of platelet 5-HT_2A_ receptor forms (monomer and dimer) and IRAS/nischarin (I_1_-IR candidate) were assessed by standard SDS-polyacrylamide gel electrophoresis (PAGE)/Western blot procedures as described [e.g., Ref. (28)]. In brief, platelet proteins (10 µg) were separated by SDS–PAGE on 10% polyacrylamide minigels (6 cm × 8 cm; Bio-Rad, CA, USA) and transferred on nitrocellulose membranes (Protran BA 85; Sigma-Aldrich, MO, USA). The membranes were incubated overnight with specific primary antibodies (Ab; dilution range 1/666 to 1/10,000) in blocking solution (PBS containing 5% nonfat-dried milk, 0.5% bovine serum albumin and 0.2% Tween20), followed by 1 h incubation with the appropriate horseradish peroxidase-conjugated secondary antibody (1/5,000 diluted anti-rabbit or anti-mouse Ab, #7074 and #7076; Cell Signaling, MA, USA). The primary Abs used were as follows: anti-5-HT_2A_ receptor Ab, #RA24288, lot: 401281 (Neuromics, MN, USA); anti-NISCH Ab, #56849, lot: GR111152 (Abcam, UK); anti-β-actin Ab, #A1978, lot: 065M4837V (Sigma-Aldrich).

**Figure 1:**
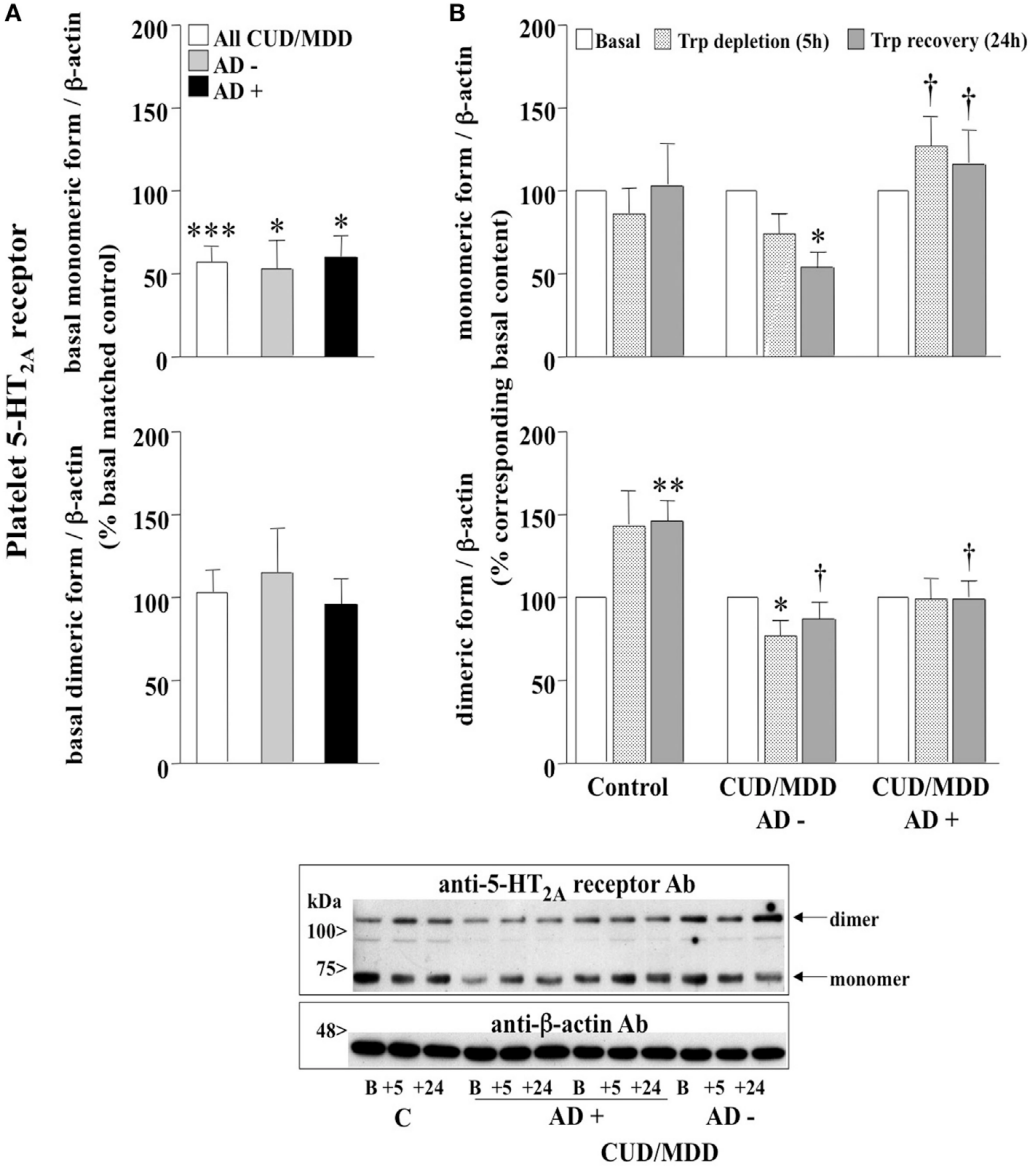
**(A)** Basal contents of platelet 5-HT_2A_ receptor mono- and dimer in antidepressant drugs (AD)-free (AD−; *n* = 7–8) and AD-treated (AD+; *n* = 10–14) comorbid cocaine use disorder (CUD)/major depressive disorder (MDD) subjects (all: *n* = 17–22). Columns are mean ± SEM of basal 5-HT_2A_ receptor contents (normalized to β-actin) in CUD/MDD groups expressed as the percentage of the corresponding basal values in matched healthy controls (control, C; *n* = 10). *p < 0.05, ***p < 0.001, when compared with the corresponding basal matched control (not reported in figure = 100%; two-tailed one-sample *t*-test). **(B)** Effect of the acute tryptophan depletion (ATD) test on the contents of platelet 5-HT_2A_ receptor mono- and dimer in healthy controls (*n* = 10), AD− (*n* = 7–8) and AD+ (*n* = 10–14) comorbid CUD/MDD subjects. Columns are mean ± SEM of 5-HT_2A_ receptor contents (normalized to β-actin) in C and CUD/MDD groups expressed as the percentage of the corresponding basal values. *p < 0.05, **p < 0.01, when compared with the corresponding basal (100%; two-tailed one-sample *t*-test); ^†^*p* < 0.05, comparing the ATD-induced alterations at 5 and 24 h in AD− and AD + CUD/MDD groups (monomeric 5-HT_2A_) or comparing the changes of dimeric receptor contents in C and CUD/MDD groups at 24 h (two-tailed unpaired Student’s *t*-test). Below: representative immunoblot for the detection of platelet 5-HT_2A_ receptor forms in control (C, B: basal value and +5 and +24 h after depletion) and AD−/+ CUD/MDD subjects (B: basal value and +5 and +24 h after depletion).

The 5-HT_2A_ receptor antibody used [validated in brain tissue of 5-HT_2A_ receptor KO mice (30)], immunodetected the platelet monomeric receptor as a ~60 kDa band and a ~120 kDa peptide which most probably represents (detected at twice the molecular mass) the homodimeric form of the 5-HT_2A_ receptor (31, 32) resistant to the denaturalizing assay conditions (see Figure 1). In addition to the possibility of 5-HT_2A_ receptor heterodimerization (31, 32), more recent studies have demonstrated that this serotonin receptor subtype can form functional homodimers resistant to the reducing agent dithiothreitol (33, 34). The IRAS/nischarin antibody has also been validated in previous studies (15, 16). Enhanced chemiluminescence detection system (Amersham, UK) was used to visualize the bound antibody on autoradiographic film (Amersham) and the resulting immunoreactive bands were quantified (integrated optical density, IOD) by densitometric scanning (GS-800 densitometer; Bio-Rad).

Comorbid CUD/MDD subjects were matched to healthy volunteers (control, C; 2–3 comorbid CUD/MDD subjects per C) (see Table 1) to assess basal differences of target proteins between these groups. In all subject groups, the platelet samples from the ATD test (5 h/Trp depletion, 24 h/Trp recovery) were compared with the corresponding subjects’ basal value in the same gel and the content (IOD) of target proteins was normalized to that of β-actin. Whenever possible, platelet samples (10 µg) for the studied subjects were assessed twice.

### Data and Statistical Analysis

The program GraphPad PRISM™, version 6.0 (GraphPad Software, CA, USA) was used for data analysis. All data sets are expressed as mean values ± SEM. Prior to statistical analyses, the parametric data were tested for normality (Kolmogorov–Smirnov normality test) and inspected for possible outliers (Grubb’s test; critical value: *Z* > 1.96; GraphPad Software at www.graphpad.com/quickcalcs/grubbs1.cfm). Detected outliers for a particular target protein were discarded from further analysis and the final number of cases (healthy controls, CUD/MDD) analyzed for target proteins is indicated in the corresponding figure legend. The experimental design of this study incorporated the pairing of comorbid CUD/MDD subjects with healthy controls, matched for different variables (mainly gender and age, see Table 1), and consequently a statistical test that takes pairing into account was performed. Analysis of covariance indicated that sex distribution (reduced number of females) and age did not influence the results (data not shown). A two-tailed one-sample *t*-test (with *N* − 1 degrees of freedom), which compares the experimental mean (percentage of control) with a hypothetical mean of 100, was used for the statistical evaluations (35). The effects of ATD on target proteins in different subject groups were evaluated by Student’s two-tailed *t*-test. The level of significance was set at *p* < 0.05 for all statistical evaluations.

## Results

### Sociodemographic, Clinical Characteristics, and the Acute Trp Depletion Test

The main sociodemographic and clinical characteristics of the sample are described in Table 1. As expected, after the induction of the ATD test, the plasma level of the amino acid Trp was markedly reduced at 5 h (>85%) in all subject groups (*p* < 0.01 for each group, Table 1).

### Platelet 5-HT_2A_ Receptor in CUD/MDD Patients: Basal Receptor Contents and Effects of ATD and AD

The basal content of monomeric 5-HT_2A_ receptor form was markedly reduced in platelets of comorbid CUD/MDD subjects (all patients: 43%), when compared with matched controls (100%), and this receptor down-regulation was independent of the presence or absence of AD medication (decreases in AD−: 47% and in AD+: 40%) (Figure 1A, upper panel). In contrast, the basal content of dimeric 5-HT_2A_ receptor form in platelets of comorbid CUD/MDD subjects, regardless of AD treatments, was not significantly different to that in control subjects (Figure 1A, lower panel).

When compared with the corresponding platelet 5-HT_2A_ receptor basal value (100%), ATD had no significant effect on monomeric receptor contents in healthy controls (5 and 24 h; Figure 1B, upper panel). In contrast, ATD further decreased 5-HT_2A_ receptor density in unmedicated comorbid CUD/MDD subjects (decreases in AD−: 26–46% at 5–24 h) (Figure 1B, upper panel), whereas AD medication was associated with an upregulation of 5-HT_2A_ monomeric receptor form (increases in AD+: 28–17% at 5–24 h; Figure 1B, upper panel).

In healthy controls, ATD increased the content of platelet 5-HT_2A_ receptor dimer (44–46% at 5–24 h; Figure 1B, lower panel). In contrast, the dimeric form of platelet 5-HT_2A_ receptor was found reduced after ATD in unmedicated comorbid CUD/MDD subjects (decreases in AD−: 23–13% at 5–24 h) (Figure 1B, lower panel). The ATD had no significant effects on the contents of 5-HT_2A_ receptor dimer in comorbid CUD/MDD subjects following AD treatment at 5–24 h (Figure 1B, lower panel).

Similar analyses were performed between patients with primary MDD or CUD-induced MDD Comparison of these two subgroups of subjects revealed no significant differences for the density of platelet monomeric and dimeric 5-HT_2A_ receptor forms at basal level and during/after ATD (data not shown).

### Platelet IRAS/Nischarin in CUD/MDD Patients: Basal Protein Content and Effects of ATD and AD

**Figure 2:**
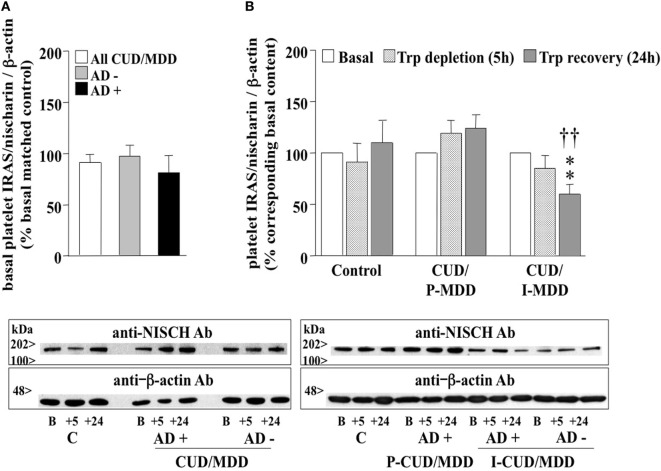
**(A)** Basal platelet imidazoline receptor antisera selected (IRAS)/nischarin contents in antidepressant drugs (AD)-free (AD−, *n* = 9) and AD-treated (AD+, *n* = 14) comorbid cocaine use disorder (CUD)/major depressive disorder (MDD) subjects (all: *n* = 23). Columns are mean ± SEM of IRAS/nischarin contents (normalized to β-actin) in CUD/MDD groups expressed as the percentage of the corresponding basal values of matched healthy controls (control, C; *n* = 8). **(B)** Effect of the ATD test on the contents of platelet IRAS/nischarin in healthy control (*n* = 8), comorbid CUD with primary MDD (CUD/P-MDD, *n* = 12–15), and CUD-induced MDD (CUD/I-MDD, *n* = 9) subjects. Columns are mean ± SEM of IRAS/nischarin contents (normalized to β-actin) in C as well as CUD/MDD groups expressed as the percentage of the corresponding basal values. **p < 0.01, when compared with the corresponding basal (100%; two-tailed one-sample *t*-test); ^††^*p* < 0.01 comparing the ATD-induced alterations at 24 h in the CUD/I-MDD group with that in the CUD/P-MDD group (two-tailed unpaired Student’s *t*-test). Below: representative immunoblots for the detection of platelet nischarin in control and AD−/+ CUD/MDD subjects.

Imidazoline receptor antisera selected/nischarin, a putative I_1_-IR, was immunodetected in human platelets as a single 167 kDa protein (Figure 2). Even though no basal differences were found for platelet IRAS/nischarin content in comorbid CUD/MDD subjects when compared with controls (100%, Figure 2A), its expression was regulated in a dissimilar manner in the different subject subgroups throughout the ATD test (Figure 2B). Compared to the corresponding basal value (100%), ATD had no effect on platelet IRAS/nischarin in the case of healthy controls but significantly reduced its expression at 24 h in CUD-induced MDD (40%) subjects (Figure 2B)., Conversely, CUD with primary MDD was associated with increased (11–24%) platelet IRAS/nischarin contents in the ATD test (Figure 2B). This discrepant regulation originated significant differences for the IRAS/nischarin contents at 24 h between the two groups of subjects with CUD, those with primary and those with CUD-induced MDD (Figure 2B). It is to note that these alterations appeared to be independent of AD treatment (data not shown).

## Discussion

The present study was designed to assess whether platelet 5-HT_2A_ receptor and IRAS/nischarin (I_1_-IR) could serve as peripheral biomarkers for comorbid CUD/MDD with a potential to discriminate between cocaine-induced and primary MDD at basal level and after ATD. The findings revealed marked reductions of platelet monomeric 5-HT_2A_ receptor protein in comorbid CUD/MDD patients at basal level, which appeared to be independent of AD treatment but without differences between primary MDD and CUD-induced MDD.

Major depressive disorder has previously been associated with increased platelet 5-HT_2A_ receptor densities in radioligand ([^3^H]- or [^125^I]-LSD) binding studies (36–38), but the effects of CUD have not been assessed so far. Assuming that the monomeric form of 5-HT_2A_ receptor is the responsible entity for antagonist binding (37, 38), the opposite regulation reported in the present study (reduced 5-HT_2A_ receptor protein *versus* increased binding sites) indicates marked basal differences of platelet 5-HT_2A_ receptor contents in primary MDD and comorbid CUD and MDD. Notably, ATD further decreased the monomeric receptor form in AD-free comorbid CUD and MDD, but not in AD-treated patients and healthy controls (Figure 1B), suggesting that AD drugs, being ineffective at modulating basal receptor contents, prevented the strengthened receptor down-regulation induced by low plasma Trp levels in comorbid CUD and MDD.

Besides similar contents of platelet 5-HT_2A_ receptor dimer in control subjects and comorbid CUD/MDD patients at basal level, the ATD increased this receptor form only in the control group and rather reduced its contents in AD-free CUD/MDD. This adaptation to low plasma tryptophan (and consequently 5-HT) levels observed in healthy controls suggests a special importance of the dimeric form in 5-HT_2A_ receptor signaling and functions in platelets. When compared with controls, the lack of response to ATD (or even the reduced contents observed in AD-free) seen in comorbid CUD/MDD patients reveals again dysregulations of the peripheral serotonergic system in this dual pathology, which may contribute to altered platelet reactivity and aggregation associated with cardiovascular disease in depression (39).

The present study also assessed the expression of the potential I_1_-IR IRAS/nischarin in platelets of CUD/MDD patients. Interestingly, although in patients they were not significantly altered at basal level, differences were found among primary or CUD-induced MDD after ATD. Patients with comorbid CUD and primary MDD showed nonsignificant increases, whereas CUD-induced MDD was associated with decreased nischarin contents during ATD. This opposite modulation suggests that platelet IRAS/nischarin could serve as a biomarker for discrimination between primary and CUD-induced MDD in comorbid CUD/MDD subjects (independent of AD medication).

The functional consequences of differential IRAS/nischarin regulation during ATD remain to be determined. Nevertheless, based on its interaction with the α5 integrin subunit of the fibronectin receptor (14), which in platelets mediates adhesion and thrombus growth (40), alterations of IRAS/nischarin contents could modulate the coagulant properties of these peripheral cells (16) and, consequently, contribute to morbid cardiovascular events reported to co-occur with CUD (41) and MDD (42).

The fact that ATD decreased IRAS/nischarin similarly in AD-free and AD-treated CUD-induced MDD, but not in AD-treated comorbid CUD/MDD with primary MDD, might reflect a poor sensitivity and responsiveness to AD treatment in CUD-induced MDD. This is relevant because at present one of the clinical challenges of comorbid depression with CUD is a lack of antidepressant treatment efficacy (43). Thus, the availability of biomarkers that can differentiate between induced and primary MDD will help to improve the treatment in this severe dual pathology.

### Limitations

Study limitations are noted. The relatively small sample size within subject groups was the main limiting factor. In this study, arising from strict recruitment criteria for patients with CUD without any other substance use disorder than nicotine (especially alcohol use disorder, which frequently coexists with CUD) and patients without active cocaine use in the last 4 weeks. The latter was very difficult to recruit mainly in the CUD-induced MDD group, which has previously been associated with higher relapse rates than Primary MDD (hazard ratios 6.5 vs 2.7 respectively) (44) emphasizing the need to improve diagnosis and appropriate treatment in these patients. Finally, sex differences in biomarkers (45) could be assessed due to the small number of female participants.

Strengths of the study are also noted. The diagnostic procedures that were used (PRISM) has better reliability and validity for drug dependence and MDD diagnoses (primary and substance induced) in comparison to other available procedures (25, 26), but its systematic use in clinical practice is difficult because it needs well trained professionals and lasts about 1–3 h to be administrated. PRISM is a good instrument for research, but not for usual clinical practice. Thus, there is a need of biomarkers that can facilitate the diagnosis in an easy and valid way. Also, these biomarkers would improve the knowledge of neurobiological mechanisms of both types of depression associated to CUD and facilitate the appropriate treatment. In addition, comparable results (Trp depletion: 86–87%) have been reported in studies using a similar protocol in pure MDD (19, 33).

## Conclusion

The main findings of the present study indicated basal reductions of monomeric 5-HT_2A_ receptor contents in platelets of comorbid CUD/MDD patients and ATD-induced dysregulations of platelet 5-HT_2A_ receptors and IRAS/nischarin. Notably, the differential modulation of platelet IRAS/nischarin during ATD, discriminating between primary and CUD-induced MDD, could be a useful biomarker to improve the diagnosis in this dual pathology. Future studies are needed to consolidate these promising results.

## Ethics Statement

The clinical protocol was approved by the local Research Ethical Committee (CEIC-Parc de Salut Mar, Barcelona, Spain), and the study was conducted in accordance with the Declaration of Helsinki and Spanish laws concerning clinical research. Healthy controls were financially compensated. All subjects gave the written, informed consent prior to participation in the study. The neurochemical protocol was also approved by the Ethics Committee of Clinical Investigation (CEIC-CAIB) and developed following the guidelines of the University of the Balearic Islands (UIB).

## Author Contributions

MT, JG-S, and MF designed the clinical and experimental protocols of the study and supervised the research. J-IM-P, DM-S, PR, FF, GV, RR-M, CT, and MT recruited the various groups of patients and matched control subjects and assessed the clinical evaluations. CP-M, EP, JM, RR-M, and J-IM-P performed the acute tryptophan depletion test sessions and the processing of blood samples, under the supervision and direction of MF. BK prepared the platelet membranes, performed the immunoblot experiments, analyzed the biochemical data, and prepared the figures with the collaboration of MA-B and MJG-F. The article was jointly written by BK, JG-S, and MT with inputs from all the co-authors.

## Conflict of Interest Statement

The authors declare that the research was conducted in the absence of any commercial or financial relationships that could be construed as a potential conflict of interest.
